# Robotic-assisted versus laparoscopic bowel anastomoses: randomized crossover in vivo experimental study

**DOI:** 10.1007/s00464-023-10044-7

**Published:** 2023-04-18

**Authors:** Caelán Max Haney, Karl-Friedrich Kowalewski, Mona Wanda Schmidt, Franziska Lang, Vasile Bintintan, Carolyn Fan, Fabian Wehrtmann, Alexander Studier-Fischer, Eleni Amelia Felinska, Beat Peter Müller-Stich, Felix Nickel

**Affiliations:** 1 Department of General, Visceral and Transplantation Surgery, Heidelberg University Hospital, Im Neuenheimer Feld 420, 69120 Heidelberg, Germany; 2 Department of Urology, University Hospital Leipzig, Liebigstraße 20, 04103 Leipzig, Germany; 3 Department of Urology, University Medical Center Mannheim, University of Heidelberg, Theodor-Kutzer-Ufer 1-3, 68167 Mannheim, Germany; 4 Department of Surgery, University Hospital Cluj Napoca, Cluj-Napoca, Romania; 5 Department of General, Visceral, and Thoracic Surgery, University Medical Center Hamburg-Eppendorf, Hamburg, Germany; 6 Department of Surgery, Clarunis University Center for Gastrointestinal and Liver Disease, University Hospital and St. Clara Hospital Basel, Basel, Switzerland

## Abstract

**Background:**

Initial learning curves are potentially shorter in robotic-assisted surgery (RAS) than in conventional laparoscopic surgery (LS). There is little evidence to support this claim. Furthermore, there is limited evidence how skills from LS transfer to RAS.

**Methods:**

A randomized controlled, assessor blinded crossover study to compare how RAS naïve surgeons (*n* = 40) performed linear-stapled side-to-side bowel anastomoses in an in vivo porcine model with LS and RAS. Technique was rated using the validated anastomosis objective structured assessment of skills (A-OSATS) score and the conventional OSATS score. Skill transfer from LS to RAS was measured by comparing the RAS performance of LS novices and LS experienced surgeons. Mental and physical workload was measured with the NASA-task load index (NASA-Tlx) and the Borg-scale.

**Outcomes:**

In the overall cohort, there were no differences between RAS and LS for surgical performance (A-OSATS, time, OSATS). Surgeons that were naïve in both LS and RAS had significantly higher A-OSATS scores in RAS (Mean (Standard deviation (SD)): LS: 48.0 ± 12.1; RAS: 52.0 ± 7.5); *p* = 0.044) mainly deriving from better bowel positioning (LS: 8.7 ± 1.4; RAS: 9.3 ± 1.0; *p* = 0.045) and closure of enterotomy (LS: 12.8 ± 5.5; RAS: 15.6 ± 4.7; *p* = 0.010). There was no statistically significant difference in how LS novices and LS experienced surgeons performed in RAS [Mean (SD): novices: 48.9 ± 9.0; experienced surgeons: 55.9 ± 11.0; *p* = 0.540]. Mental and physical demand was significantly higher after LS.

**Conclusion:**

The initial performance was improved for RAS versus LS for linear stapled bowel anastomosis, whereas workload was higher for LS. There was limited transfer of skills from LS to RAS.

**Supplementary Information:**

The online version contains supplementary material available at 10.1007/s00464-023-10044-7.

## Background

Since the introduction of laparoscopic surgery (LS), it has gained strong acceptance in the surgical field. Not only operations for benign indications such as appendicitis or cholecystitis are now performed laparoscopically but also major oncologic operations such as colectomy and major liver resections have shown to have the benefits of the laparoscopic approach—lower blood loss, faster recovery, and shorter hospital stay [1–5].

The share of operations that are being performed with robotic assistance has increased during the last 20 years [6]. While initially, robotic-assisted surgery (RAS) was broadly used mainly in urologic surgery, nowadays many general surgeons have taken up the technique. While some surgeons are directly switching from open surgery to RAS without previous experience in conventional LS, many experienced laparoscopic surgeons are also changing their approach to RAS. Reasons for this technique migration are promises of increased technical ability to perform difficult reconstructive tasks, such as gastrointestinal anastomoses, an advanced but general skill that is necessary in many operations in general surgery, urinary diversion in urology and also in gynecological surgery. RAS is expected to lower the learning curve as compared to conventional laparoscopy, [7] a concept that is widely discussed whenever new surgical techniques are introduced. However, there is limited evidence that the potential for increased technical skills or shorter learning curves is fulfilled.

In a previous study, we were not able to show that laparoscopic basic technical skills transfer to RAS in a simulated surgical environment [8]. The same was shown for previous experience with open surgery. As multiple studies have shown that technical skills in a virtual environment might predict skills in an operating room setting [9], this begs the question if skills learned in LS transfer to another minimally invasive entity such as RAS or to which extent the surgeons have to undergo another learning curve.

Goal of the present study was to examine if the initial learning curve for reconstructive tasks is faster with RAS than with conventional laparoscopy for inexperienced surgeons in an in vivo model. Furthermore, the skill transfer between laparoscopy and RAS was examined by comparing skills between experienced surgeons in laparoscopy that are naïve robotic surgeons and surgeons that are naïve in both LS and RAS. In addition, the differences in physical and mental workload between the two approaches were assessed.

## Methods

### Trial design

A randomized, outcome assessor blinded, crossover trial that compared the initial learning curve of laparoscopic and robotic-assisted side-to-side anastomoses with hand-sutured closure of the remaining enterotomy was performed in an in vivo porcine model. The CONSORT guidelines for randomized controlled crossover trials were adhered to [10].

### Participants and setting

**Fig. 1:**
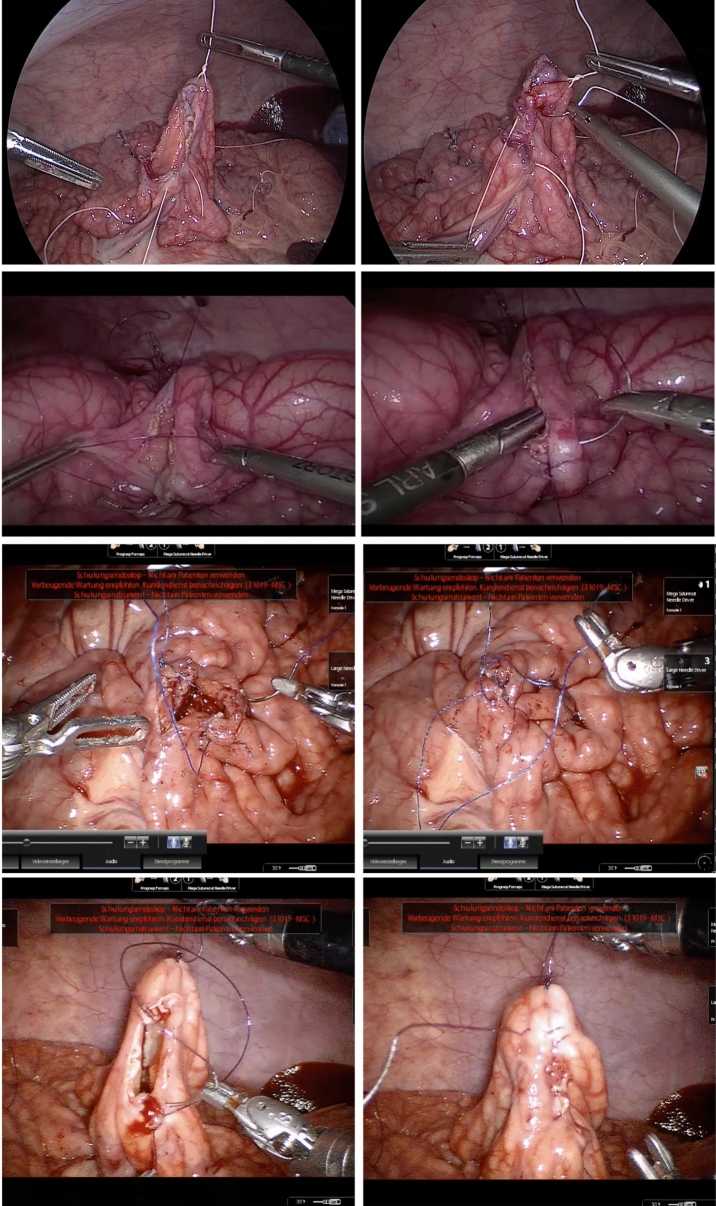
First row: Laparoscopic anastomosis with low A-OSATS score; Second row: Laparoscopic anastomosis with higher A-OSATS score; Third row: Robotic-assisted anastomosis with low A-OSATS score; Fourth row: Robotic-assisted anastomosis with higher A-OSATS score

Surgeons from the Department of General, Visceral and Transplant Surgery at Heidelberg University were recruited. Ethics approval from the local ethics committee was attained (S-436/2018). The surgeons had varying experience in LS and no to very little experience in RAS. For LS, surgeons were classified as “novices” if they had performed 0 to 100 procedures of the respective technique. Surgeons that had performed more than 100 procedures were classified as experienced surgeons. This classification as “experienced” was based on a systematic review of laparoscopic roux-en-Y gastric bypass. [11]. For open surgery, surgeons were classified as novices if they had performed less than 100 procedures, as experienced surgeons if they had performed more than 100 procedures (also see Fig. 1 for intraoperative setup). A maximum of 4 weeks was set as a wash-out period so that surgeons were not able to gain extensive experience in either technique between the tries.

### Interventions

A standardized technique of side-to-side stapled anastomoses with hand sutured closure of the remaining enterotomy was demonstrated to the surgeons with the help of a video and a 3-d printed model (technique shown in Fig. 1). After this, the surgeons were randomized into two groups in a 1:1 fashion with the help of an online randomization tool using opaque randomization envelops. The first group initially performed a laparoscopic anastomosis in the demonstrated fashion. After this, the surgeons performed a robotic-assisted anastomosis using the same technique. All anastomoses were performed on a live porcine model. The second group initially performed a robotic-assisted anastomosis and then a laparoscopic anastomosis using the same technique.

### Outcomes

The primary outcome was the comparison between the technical skill of the laparoscopic and robotic-assisted anastomosis for novice laparoscopic surgeons. The quality of the surgical technique was measured using a previously validated score for side-to-side, linear-stapled anastomoses with hand-sutured closure of the enterotomy, the anastomosis objective assessment of technical skills (A-OSATS) score. [12] The A-OSATS score sections the creation of a side-to-side stapled anastomosis into 4 distinct steps (1: bowel placement and setup, 2: creation of enterotomies; 3: stapling; 4: closure of enterotomies), that consist of different substeps. Each substep is rated with the help of pointers. The final score indicates how well the anastomosis was performed.

Secondary outcomes were the assessment of skill transfer of expert laparoscopic surgeons from laparoscopy to RAS by comparing the skills in RAS with those of novice laparoscopic surgeons. Additionally, the mental and physical workload of both novices and experienced surgeons in both surgical techniques was assessed. Additional endpoints were time and the individual subscales of the A-OSATS. Surgeon perceived workload was assessed by the NASA task load index (NASA-TLX), a validated scoring instrument in which 6 different domains (mental demand, physical demand, temporal demand, performance, effort, and frustration) are assessed on a scale of 0–100 in steps of five. Physical stress was measured using the German version of the Borg CR10 scale by each surgeon before and after each set of anastomoses. [13–15]. For the Borg-scale, surgeons rate the physical stress on different body parts using a scale of 0–10 with the help of text prompts.

Rating was performed by an experienced rater that rated each individual anastomosis using the captured video data. The rater was blinded to the experience level of the surgeons and the randomization group.

### Sample size

The sample size was based on a pragmatic decision to include as many surgeons as possible into the trial. Due to the available number of pigs, with the calculation being that about 3–5 anastomoses could be viably performed on one pig, the final sample size was determined. In addition, this number would provide reasonable effect estimates that can serve as basis for future trials.

### Statistical Analysis

All analyses were carried out with the R Software (Version 3.6.2.9). [16] Continuous outcomes were summarized with the mean ± standard deviation (SD) for parametric data and with the median (interquartile range) in the case of non-parametric data. For categorical data, absolute and relative frequencies were given. For group comparisons, paired test were used as during the crossover trial, each subject served as its own comparator. Thus, the paired *t *test was used for parametric data, and the Wilcoxon signed rank test for non-parametric data. An alpha value ≤ 0.05 of was considered statistically significant.

### Results

In total, forty surgeons were included in the trial. Baseline characteristics for the randomized comparison of the initial learning curve and the analysis of experienced surgeons versus Novices can be found in Table 1.

**Table 1:** Baseline overview for the entire cohort and the subgroups “novices” and “experienced surgeons”

Overall cohort	Overall	Novices	Experienced surgeons	*p *Value
*n*	40	24	16	
Age [mean (SD)]	33.8 ± 6.9	29.8 ± 3.2	39.4 ± 7.0	< 0.001
With experience in open surgery *n* (%)	23 (57.5)	7 (29.2)	16 (100.0)	< 0.001
With experience in conventional laparoscopic surgery *n* (%)	16 (40.0)	0 (0.0)	16 (100.0)	< 0.001
With experience in robotic surgery *n* (%)	0 (0.0)	0 (0.0))	0 (0.0)	–

#### Operative performance

As planned in the study protocol, the overall cohort was compared as randomized controlled crossover comparison between the robotic and laparoscopic approach. There were no significant differences, except for time needed to close the enterotomy favoring the laparoscopic approach (Table 2).

**Table 2:** Comparison of operative performance between laparoscopic and robotic anastomoses during the early learning curve (All values are given as mean ± standard deviation)

	Overall Cohort (n = 40)				Laparoscopic novices (*n* = 24)			
Surgical parameters	Laparoscopic anastomosis	Robotic anastomosis	*P *Value*	MD [95% CI]	Laparoscopic anastomosis	Robotic anastomosis	*P* Value*	MD [95% CI]
Full A-OSATS	51.1 ± 12.1	52.3 ± 7.7	0.414	1.2 [− 2.1; 4.5]	48.0 ± 12.1	52.0 ± 7.5	**0.044**	**4.0 [0.1; 8.0]**
A-OSATS—bowel positioning	9.0 ± 1.3	9.3 ± 0.9	0.091	0.3 [− 0.1; 0.7]	8.7 ± 1.4	9.3 ± 1.0	**0.045**	**0.6 [0.0; 1.1]**
A-OSATS—creation of enterotomy	11.9 ± 2.4	11.8 ± 2.2	0.955	− 0.03 [− 0.9; 0.0]	11.4 ± 2.7	11.6 ± 2.1	0.791	0.2 [− 1.2; 1.5]
A-OSATS—stapling	15.9 ± 4.6	15.5 ± 3.1	0.608	− 0.4 [− 1.9; 1.1]	15.0 ± 4.7	15.6 ± 3.1	0.558	0.6 [− 1.4; 2.5]
A-OSATS—closure of enterotomy	14.4 ± 5.9	15.7 ± 4.4	0.119	1.3 [− 0.4; 3.0]	12.8 ± 5.5	15.6 ± 4.7	**0.010**	**2.7 [0.7; 4.8]**
Full Time in min	25.3 ± 9.3	26.5 ± 8.8	0.478	1.2 [− 2.2; 4.6]	27.6 ± 9.8	28.3 ± 9.2	0.797	0.7 [− 4.5; 5.8]
Time in sec—bowel positioning	294 ± 174	279 ± 128	0.649	− 15 [− 83; 53]	326 ± 191	298 ± 150	0.602	− 28 [− 138; 82]
Time in sec—creation of enterotomy	121 ± 58	103 ± 48	0.129	− 18 [− 41; 5]	113 ± 45	106 ± 54	0.627	− 7 [− 37; 22]
Time in sec—stapling	230 ± 119	203 ± 112	0.194	− 28 [− 70; 15]	253 ± 130	225 ± 131	0.392	− 28 [− 94; 38}
Time in sec—closure of enterotomy	906 ± 333	1037 ± 390	**0.033**	**130 [11; 250]**	995 ± 335	1100 ± 371	0.211	105 [− 64; 274]

*A-OSATS* Anastomosis Objective Structured Assessment of Technical Skills; *paired *t *test, *MD* Mean Difference; *CI* Confidence interval

Significant at *p*≤0.05 are shown in bold

**Fig. 2:**
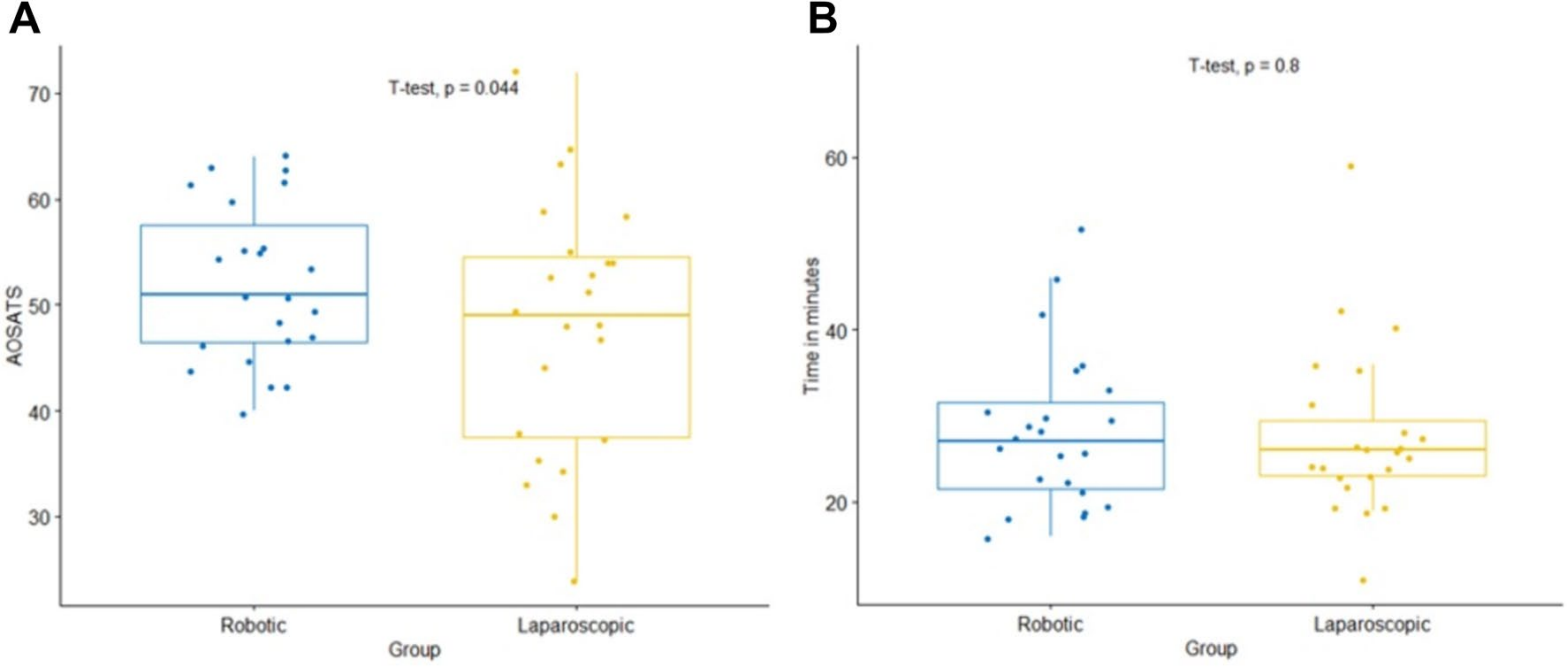
**A** Comparison of operative performance (A-OSATS) and **B** operating time between robotic and laparoscopic anastomosis of novices

As the missing effect derived most likely from the group of experienced laparoscopists, a post-hoc sub-group analysis considering novices only was performed. Here, surgeons performed significantly better when using the robotic system in terms of overall performance (A-OSATS), bowel positioning and closure of enterotomy. There was no evidence for a difference between the two approaches for operation time needed (Table 2). Main outcomes such as A-OSATS and operating time are visualized in Fig. 2 for the laparoscopic group and in Supplementary Fig. 2 for the overall cohort.

Additionally, there was no difference in overall performance considering the order of training (robotic first or laparoscopic first—see Supplementary Table 1).

#### Transferability of skills from laparoscopic to robotic surgery

While experienced surgeons outperformed Novices for the laparoscopic anastomosis for relevant parameters, no relevant differences were observed for the robotic anastomosis (Table 2).

#### Physical and psychological stress

Comparing physical and psychological demand, it was found that the laparoscopic approach caused significantly more discomfort in the upper body. Additional, as measured by the NASA TLX score, LS was associated with a higher level of physical demand, effort, and frustration (Table 3).

**Table 3:** Comparison of physical and psychological demand between laparoscopic and robotic surgery [All values are given as median (interquartile range)]

Domain	Laparoscopic anastomosis	Robotic anastomosis	*p *Value*
Borg scale—neck	0.8 (0–2)	0 (0–1)	**0.002**
Borg scale—trapezoid	0.5 (0–1)	0 (0–0)	**0.012**
Borg scale—shoulder right	0 (0–2)	0 (0–0.5)	**0.038**
Borg scale—shoulder left	0 (0–1)	0 (0–0.5)	**0.047**
Borg scale—forearm right	0 (0–0.5)	0 (0–0)	0.079
Borg scale—forearm left	0 (0–0.5)	0 (0–0)	0.724
Borg scale—hand right	0 (0–0.5)	0.2 (0–1)	0.570
Borg scale—hand left	0.5 (0–2)	0.5 (0–2)	0.665
Borg scale—back	0 (0–1)	0 (0–1)	0.098
Borg scale—leg right	0 (0–0)	0 (0–0)	0.938
Borg scale—leg left	0 (0–0.1)	0 (0–0)	0.795
NASA TLX—mental demand	45 (29–66.2)	50 (20–70)	0.809
NASA TLX—physical demand	57.5 (40–70)	30 (20–45)	** < 0.001**
NASA TLX—temporal demand	40 (30–60)	40 (20–51.2)	0.147
NASA TLX—performance	52.5 (30–70)	55 (30–70)	0.713
NASA TLX—effort	67.5 (50–76.2)	60 (40–70)	**0.024**
NASA TLX—frustration	40 (20–70)	25 (14–60)	**0.025**

*Wilcoxon signed rank test

Significant at *p*≤0.05 are shown in bold

## Discussion

This randomized controlled, outcome assessor blinded crossover study was able to demonstrate that the initial performance for creating minimally invasive linear-stapled intestinal anastomoses is better with the robotic-assisted approach compared to conventional laparoscopy. Furthermore, the study shows that previously acquired laparoscopic skills do not transfer to RAS. Physical workload, and frustration were lower in RAS.

Even though RAS has shown only few advantages in randomized controlled clinical trials, its share has increased exponentially in the last years [6]. As was shown for laparoscopic cholecystectomy with the rise of bile duct injuries at the end of the twentieth century, the initial uptake of a new surgical technique can lead to an increase of complications. [17–19] Therefore, it is important to know which skills can be transferred from laparoscopy to RAS and which do not. This study shows that the included laparoscopic experienced surgeons had similar A-OSATS scores as novice laparoscopic surgeons when performing robotic-assisted bowel anastomoses. This leads to the conclusion that laparoscopically experienced surgeons were not able to transfer their reconstructive skills to RAS. This lack of skill transfer was previously shown in laparoscopic prostate surgery. In one such study, surgeons that had previously performed open radical prostatectomy showed an absolute risk increase of 12.3% in terms of 5-year recurrence rates when compared to surgeons that performed their first radical prostatectomy laparoscopically. [20]

Likely, this lack of a difference between the two examined groups may also be a result of the faster learning curve for RAS that was shown for novice surgeons. This was especially visible in the substep of the A-OSATS “closure of enterotomy,” which largely consists of suturing and knot tying. The underlying reason for this is probably the more intuitive handling of RAS instruments that have seven degrees of freedom. This is in line with the hypothesis that the robotic approach only adds benefits for procedures with a complex reconstruction task such as pancreaticoduodenectomy or radical cystectomy.

A further important observation in this study is the difference in the NASA TLX subscales. Surgeons reported lower levels of physical demand, less frustration and found that they needed to work less hard to create the anastomosis when operating with robotic assistance. These differences appear logical as the instruments can be operated more intuitively and the 3D vision lowers the mental demand. Additionally, surgeons reported elevated upper body discomfort after operating laparoscopically. This is especially important as the physical strain of operating laparoscopically is very high due to frequent unergonomic body positioning [21–23]. The seated position at the console might significantly lower this strain and prolong the operating lifetime of surgeons.

### Limitations

Measuring technical skills in surgery is a difficult task and has been extensively discussed since the conception of the OSATS score. Similarly, the A-OSATS was constructed by a modified Delphi expert consensus with experienced minimally invasive surgeons and afterward validated on a porcine model [12]. An alternative to using these scores would have been performing only one anastomosis per pig and measuring the rate of anastomotic insufficiencies postoperatively. This would have not been ethically possible as an immense number of pigs would have been needed to fulfill the sample size calculation. Therefore, it was decided that using the previously validated score was likely the best solution for this study. Another limitation of this study is the side-to-side stapled technique of the anastomosis with sutured closure of the enterostomy. This technique is decidedly of lower technical difficulty that comparable fully sutured techniques. Therefore, it might be stipulated that by using this technique the full potential of robotic assistance is not shown. However, especially for novices, even the stapled technique represents a very advanced skill. Using a fully sutured technique would have likely resulted in much longer operating times and might have resulted in many participants not being able to complete the given task. Lastly a limitation that must be named is the previous experience in open surgery. Surgeons that had experience in laparoscopic surgery were also more experienced in open surgery. This might have influenced the results; however, this was unavoidable due to the hospital system in which surgeons are initially trained in open surgery and subsequently in laparoscopic surgery.

## Strengths

This study included a blinded assessment of the technical skills, thereby lowering the possible bias that could be present when rating surgeons in a non-blinded live fashion as surgeon behavior could influence the rating. All technical skills scores used were previously validated and all scores to indicate physical and mental workload are well established. A large sample size was able to be collected at an experienced tertiary university center, thereby providing good generalizability.

## Conclusion

RAS improves initial performance of linear-stapled bowel anastomosis compared to LS, whereas LS led to a higher physical and mental demand for surgeons. The lower ergomic and mental demand might lead RAS surgeons to be able to operate longer in their life without the same strain as LS. Previous conventional laparoscopic experience did not influence robotic performance, thereby indicating that there is no necessity of training LS before RAS. If these data can be transferred to other reconstructions such as vessel reconstruction and biliary or pancreatic anastomoses has to be assessed. Surgeons should be offered vigorous training of robotic skills regardless of prior experience.

## Supplementary Information

Below is the link to the electronic supplementary material.

Supplementary file1 (JPG 425 KB)—Supplementary figure 1: Trial Flow Chart

Supplementary file2 (JPG 38 KB)—Supplementary Figure 2 Comparison of operative performance (A-OSATS) and B operating time between robotic and laparoscopic anastomosis of the overall cohort

Supplementary file3 (DOCX 15 KB)—Supplementary Table 1: Comparison of total performance between groups stratified by first surgical approach*paired t-test
