# Supplementary figures and images for: Robotic-assisted versus laparoscopic bowel anastomoses: randomized crossover in vivo experimental study
Source: Surg Endosc. 2023 Apr 18;37(8):5894–901. doi: 10.1007/s00464-023-10044-7 (PMC10338398; doi:10.1007/s00464-023-10044-7)

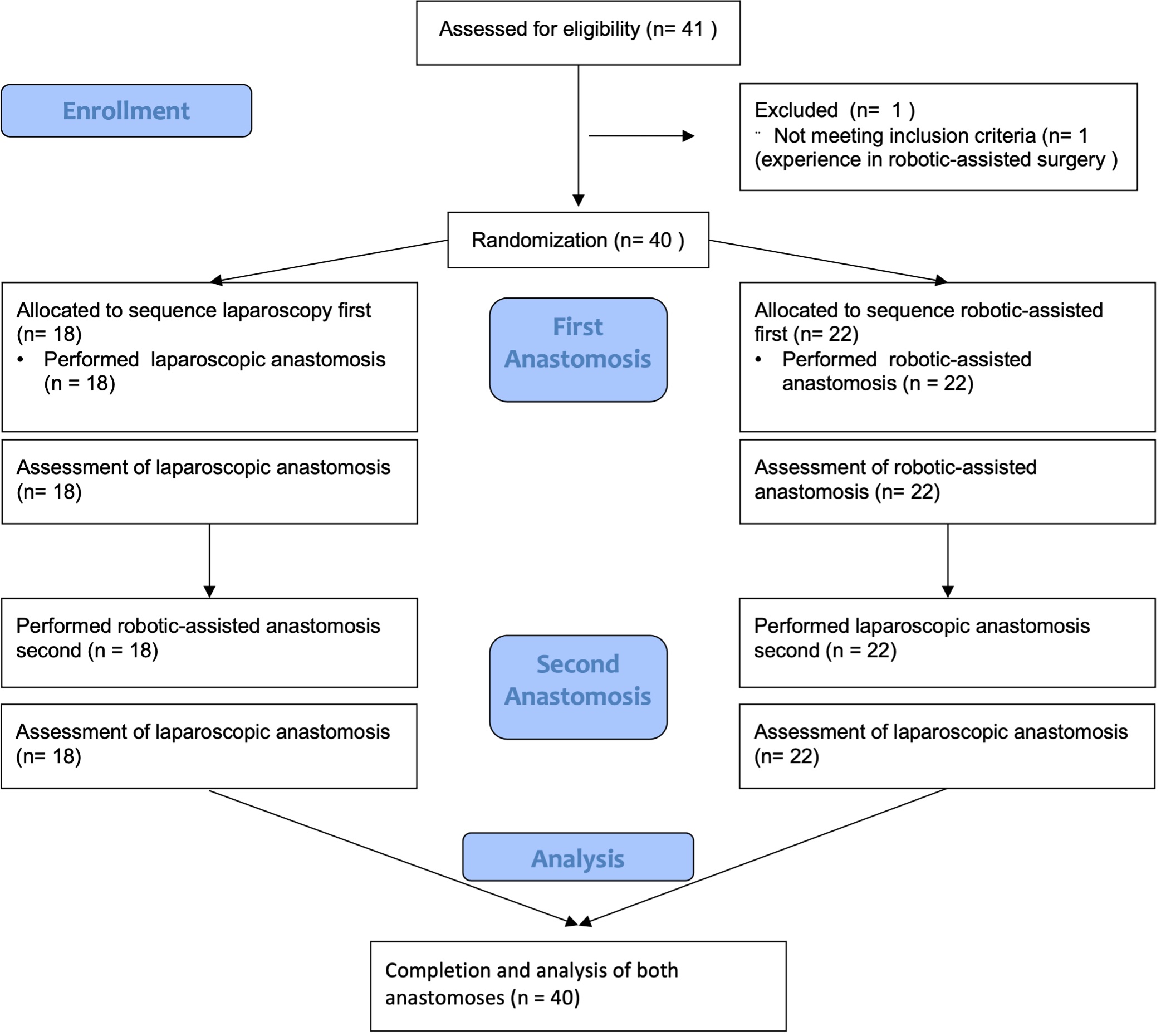

Supplement: Supplementary file 1 — Supplementary file1 (JPG 425 KB)—Supplementary figure 1: Trial Flow Chart [file 464_2023_10044_MOESM1_ESM.jpg]

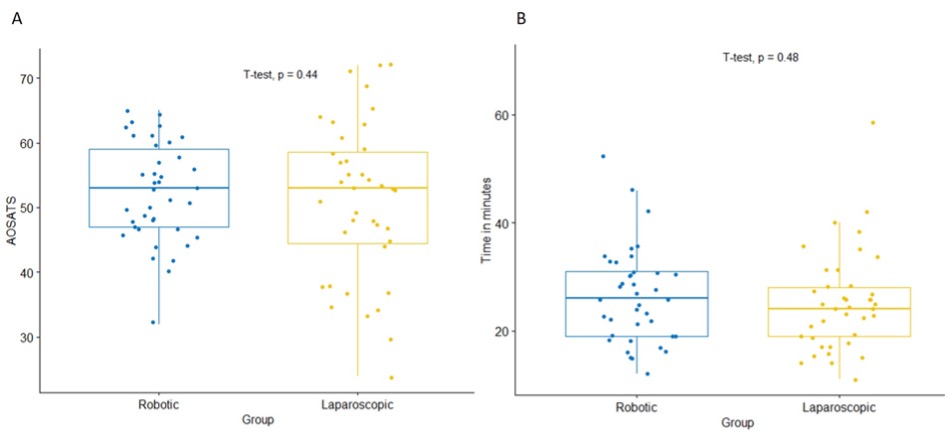

Supplement: Supplementary file 2 — Supplementary file2 (JPG 38 KB)—Supplementary Figure 2 Comparison of operative performance (A-OSATS) and B operating time between robotic and laparoscopic anastomosis of the overall cohort [file 464_2023_10044_MOESM2_ESM.jpg]
