# Supplementary material for: Robotic-assisted versus laparoscopic bowel anastomoses: randomized crossover in vivo experimental study
Source: Surg Endosc. 2023 Apr 18;37(8):5894–901. doi: 10.1007/s00464-023-10044-7 (PMC10338398; doi:10.1007/s00464-023-10044-7)
Supplement: Supplementary file 3 — Supplementary file3 (DOCX 15 KB)—Supplementary Table 1: Comparison of total performance between groups stratified by first surgical approach*paired t-test [file 464_2023_10044_MOESM3_ESM.docx]

Supplementary Table 1: Comparison of total performance between groups stratified by first surgical approach*paired t-test

|  | *Overall performance by first approach* | | |
| --- | --- | --- | --- |
| *Parameter* | *Robotic-assisted* | *Laparoscopic* | *p-Value** |
| Full A-OSATS | 104.7 ± 18.5 | 94.0 ± 16.2 | 0.162 |
| A-OSATS - bowel positioning | 17.9 ± 2.1 | 18.3 ± 2.0 | 0.607 |
| A-OSATS - creation of enterotomy | 22.9 ± 4.4 | 23.0 ± 2.6 | 0.961 |
| A-OSATS - stapling | 32.5 ± 6.3 | 28.2 ± 6.1 | 0.112 |
| A-OSATS - closure of enterotomy | 31.4 ± 9.3 | 24.5 ± 7.6 | 0.071 |
| Full Time in min | 54.3 ± 13.2 | 57.9 ± 17.2 | 0.575 |
| Time in sec - bowel positioning | 591 ± 171 | 667 ± 296 | 0.445 |
| Time in sec - creation of enterotomy | 225 ± 60 | 213 ± 86 | 0.700 |
| Time in sec - stapling | 459 ± 194 | 502 ± 240 | 0.642 |
| Time in sec - closure of enterotomy | 2051 ± 601 | 2153 ± 600 | 0.690 |
